# Continuous flow production of concentrated hyperpolarized xenon gas from a dilute xenon gas mixture by buffer gas condensation

**DOI:** 10.1038/s41598-017-07695-7

**Published:** 2017-08-04

**Authors:** Hirohiko Imai, Hironobu Yoshimura, Atsuomi Kimura, Hideaki Fujiwara

**Affiliations:** 1 Graduate School of Medicine, Osaka University, Osaka, 565-0871 Japan; 2 Graduate School of Informatics, Kyoto University, Kyoto, 606-8501 Japan

## Abstract

We present a new method for the continuous flow production of concentrated hyperpolarized xenon-129 (HP ^129^Xe) gas from a dilute xenon (Xe) gas mixture with high nuclear spin polarization. A low vapor pressure (i.e., high boiling-point) gas was introduced as an alternative to molecular nitrogen (N_2_), which is the conventional quenching gas for generating HP ^129^Xe via Rb-Xe spin-exchange optical-pumping (SEOP). In contrast to the generally used method of extraction by freezing Xe after the SEOP process, the quenching gas separated as a liquid at moderately low temperature so that Xe was maintained in its gaseous state, allowing the continuous delivery of highly polarized concentrated Xe gas. We selected isobutene as the candidate quenching gas and our method was demonstrated experimentally while comparing its performance with N_2_. Isobutene could be liquefied and removed from the Xe gas mixture using a cold trap, and the concentrated HP ^129^Xe gas exhibited a significantly enhanced nuclear magnetic resonance (NMR) signal. Although the system requires further optimization depending on the intended purpose, our approach presented here could provide a simple means for performing NMR or magnetic resonance imaging (MRI) measurements continuously using HP ^129^Xe with improved sensitivity.

## Introduction

Nuclear magnetic resonance (NMR) of hyperpolarized xenon-129 (HP ^129^Xe) atoms has been widely used in a variety of research areas, such as physics, chemistry, material science, and biomedical imaging^1, 2^. HP ^129^Xe gas is usually produced via a spin-exchange optical-pumping (SEOP) method, in which a hyperpolarized nuclear spin state is achieved based on the transfer of the angular momentum carried by photons from circularly polarized laser light^3, 4^. For this transfer process, an alkali metal (typically rubidium, Rb) is used as an intermediate. One of the two spin states of the valence electron of a vaporized Rb atom becomes populated abundantly by absorbing circularly polarized laser light with a wavelength matched to the Rb D1 transition (~795 nm). Through subsequent Rb-Xe interactions, such as binary collision between a spin-polarized Rb atom and a Xe atom or the formation of an Rb/Xe van der Waals molecular complex, the transfer of spin polarization from a Rb electron to the ^129^Xe nuclei occurs.

In order to obtain highly polarized ^129^Xe gas, two types of buffer gases are generally added to the Xe gas to promote efficient SEOP. The first, and the most important for optical pumping is to add a quenching gas such as molecular nitrogen (N_2_), which prevents the excited state of Rb atoms from re-radiating unpolarized photons, enabling the avoidance of radiation trapping. Nitrogen has a large quenching cross section^5, 6^ and a moderate spin destruction cross section for Rb (${k}_{{\rm{SD}}}^{{\rm{Rb}}-{{\rm{N}}}_{2}}$ = 1.3 × 10^−25^ *T* ^3^ cm^3^ s^−1^ ^7^, where *T* is the temperature in K) and, therefore, it is typically used as a quenching gas in SEOP, where several tens of kPa of N_2_ are added to Xe gas to provide sufficient quenching effect. A second buffer gas is added in order to broaden the absorption line of Rb to better match the spectral profile of the laser^8^. Generally, several hundreds of kPa of helium (He) are added to the Xe/N_2_ gas mixture for this purpose. Helium is chosen because it has the smallest spin destruction cross section for Rb (${k}_{{\rm{SD}}}^{{\rm{Rb}}-{\rm{He}}}$ = 1.0 × 10^−29^ *T* ^4.26^ cm^3^ s^−1^ ^9^) and, therefore, causes little loss of Rb spin polarization while broadening. Xe, in contrast, has a large spin destruction cross section for Rb (${k}_{{\rm{SD}}}^{{\rm{Rb}}-{\rm{Xe}}}$ = 6.3 × 10^−17^(*T*-273.15)^1.17^ cm^3^ s^−1^ ^10^) and, therefore, causes a significant loss of Rb spin polarization in condition with higher Xe number density, leading to a reduction in ^129^Xe nuclear spin polarization, *P*_Xe_. Based on these characteristics, a highly diluted Xe gas mixture composed of a small amount of Xe and N_2_, and a large amount of He gas has been used for generating highly polarized ^129^Xe gas^8, 10–13^.

The buffer gases needed for SEOP cause a side-effect of signal reduction through dilution of the Xe gas. By separating the HP ^129^Xe gas from the buffer gases, HP ^129^Xe gas can be concentrated. Because Xe freezes well above the boiling point of both N_2_ and He, the buffer gases can be removed by freezing out the Xe. At present, the only effective way to concentrate HP ^129^Xe for further use in NMR or magnetic resonance imaging (MRI) is freeze-thaw separation, in which Xe in the continuously flowing gas mixture is frozen and accumulated until it reaches a sufficient amount for use, and then the solid Xe is thawed to return it to a gaseous state^8, 10, 11, 14, 15^. The freeze-thaw approach, however, has some drawbacks, such as the accumulation of a sufficient quantity of solid HP ^129^Xe taking a considerable time (e.g., several tens of minutes). Although the longitudinal relaxation time *T*_1_ of solid ^129^Xe at liquid N_2_ temperature in the presence of a magnetic field is reasonably long for accumulation^16^, it often results in an extra significant loss of *P*_Xe_ in the process of solidification, accumulation, and volatilization of Xe, and this process complicates the system and its operation. Furthermore, this approach is not compatible with the continuous flow production of HP ^129^Xe gas because the Xe gas has to be stored in a solid state. Therefore, its use is limited to NMR or MRI experiments conducted in a batch mode.

Depending on the purpose, it is possible to forgo the cryogenic accumulation step to directly deliver highly or moderately dilute HP ^129^Xe mixtures to the target by continuous flow^12, 17, 18^ or stopped flow^19–23^. However, it is obvious that the use of fully concentrated ^129^Xe gas from highly dilute HP ^129^Xe mixture enables MRI applications with significantly increased signal intensity^12^. Most recently, Meersmann and coworkers demonstrated that molecular hydrogen (H_2_) can be used as a quenching gas in SEOP and it can be reactively removed via catalytic combustion for the purpose of purifying HP ^83^Kr as well as HP ^129^Xe after SEOP^24^. However, the continuous flow production of purified HP noble gas, to the best of our knowledge, has not been demonstrated previously.

The aim of this work is to develop a new method to concentrate HP ^129^Xe gas from a dilute Xe gas mixture using a new buffer gas for SEOP that is capable of continuously delivering highly spin polarized undiluted ^129^Xe gas for use in performing NMR or MRI measurement continuously with improved sensitivity. We reported a study on pulmonary functional imaging in mice using HP ^129^Xe generated using a system based on the concept described here^25^. In the present study, we focus on the basic aspects of the methodology based on a detailed analysis of Xe polarization, Rb polarization and Rb-Xe spin exchange process in the use of a new quenching gas comparing them with the conventional N_2_. Furthermore, we investigate the current shortcoming to improve the system by analyzing observed enhancement of MR signal strength and loss of polarization encountered in a concentration process of dilute Xe gas.

## Results and Discussion

### Selection of a quenching gas

Our proposal for concentrating HP ^129^Xe gas from a dilute Xe gas mixture was to use a high boiling point gas as a quenching gas that condenses at a higher temperature than Xe. In order to achieve this objective, the candidate quenching gas requires sufficient quenching ability, a higher boiling point than Xe, a small contribution to the spin destruction of Rb, and low chemical reactivity with highly reactive Rb. To quench the fluorescent emission, it is required to transfer the excitation energy of Rb atoms to kinetic energy of the quenching gas. Molecular gases with a chemical double bond possess a large quenching cross section and can effectively quench fluorescence through energy transfer into its vibrational mode^6^. Among the previously reported data regarding quenching cross section, a hydrocarbon gas ethylene (ethene, H_2_C=CH_2_) has a larger quenching cross section (*σ*(*P*_1/2_ → *S*_1/2_) = 139 Å^2^) than that of N_2_ (*σ*(*P*_1/2_ → *S*_1/2_) = 58 Å^2^)^5, 6^, and has a slightly higher boiling point (169.5 K) than Xe (165.0 K). Ethylene, however, was not suitable for our purpose because of the very small difference in boiling point, and, therefore, a gas with a substantially different boiling point was required in order to enhance the separation efficiency from Xe gas. In the present study, isobutene (2-methylpropene, H_2_C=C(CH_3_)_2_) was selected as the first candidate quenching gas because its boiling point is 266.2 K, which is over 100 K higher than Xe, and it has a chemical double bond favorable for effective quenching similar to N_2_ and ethylene.

### Continuous flow production of concentrated HP ^129^Xe gas by isobutene condensation

**Figure 1:**
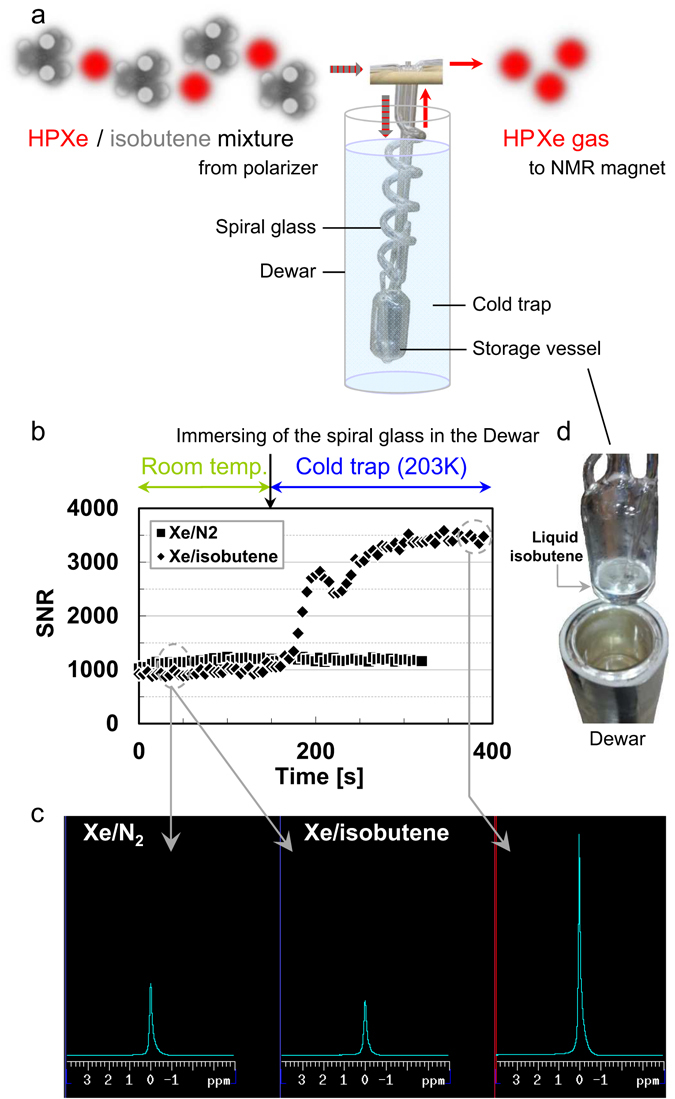
Dynamics of ^129^Xe NMR signal enhancement by removing isobutene. (**a**) Diagram of the glassware for condensing and storing isobutene using a cold trap and schematic diagram of the production of concentrated HP ^129^Xe gas from the Xe/isobutene gas mixture. (**b**) Time course of SNR of ^129^Xe spectrum using gas mixtures with Xe/isobutene (diamonds) and Xe/N_2_ (rectangles). Spiral glass piping was immersed in a Dewar at the time point indicated by the black allow to trap isobutene at 203 K. The Xe fraction in both mixtures was *f*_Xe,SEOP_ = 0.20. Gas flow rates of Xe, isobutene, and N_2_ were *F*_Xe_ = 17, *F*_ib_ = 69 and ${F}_{{{\rm{N}}}_{2}}$ = 69 sccm, respectively. (**c**) HP ^129^Xe NMR spectrum acquired from a Xe/N_2_ gas mixture (left) and a Xe/isobutene gas mixture recorded before (center) and after separation of isobutene using the cold trap (right). (**d**) Photograph of the liquid isobutene separated from Xe gas and stored in the storage vessel.

In order to demonstrate that Xe was concentrated continuously from the dilute Xe gas mixture, the ^129^Xe NMR signal was measured repeatedly from the continuously flowing Xe/isobutene gas mixture before and after condensing of isobutene. The Xe/isobutene gas mixture was flowed through a polarizer^26^ for hyperpolarization, a tubular spiral glassware as a cold trap (Fig. 1a), and an NMR tube in a 9.4-T superconducting magnet for measuring the ^129^Xe NMR signal. More details about the experimental set-up and NMR measurement are given in Methods section. Figure 1b shows a time course of the ^129^Xe signal-to-noise ratio (SNR) of the NMR spectrum measured during the continuous flow production of HP ^129^Xe using a binary gas mixture of Xe/isobutene before and after the concentration of HP ^129^Xe by application of the cold trap. Results from the Xe/N_2_ mixture are shown for comparison. For a dilute Xe/isobutene mixture without a cold trap, a stable HP ^129^Xe signal was observed continuously, although the SNR was slightly lower than that of the Xe/N_2_ mixture. For repeated acquisitions of ^129^Xe spectra, the spiral glass was put in a cold Dewar at after 150 s. Immediately after this, the ^129^Xe signal intensity began to increase, then increased further gradually with some fluctuations, and finally reached a steady state. The enhanced ^129^Xe signal continued to be measured with sufficient stability similar to the Xe/N_2_ mixture. Representative NMR spectra showing spectral enhancement by concentrating Xe are shown in Fig. 1c. After the measurement, it was confirmed that liquid isobutene was stored in the storage vessel attached to the lower side of the spiral glassware (Fig. 1d), and, therefore, isobutene had been removed from the Xe gas, and the concentrated Xe gave the enhanced signal.

### Comparison between isobutene and N_2_ as a quenching gas for Rb-Xe SEOP

**Figure 2:**
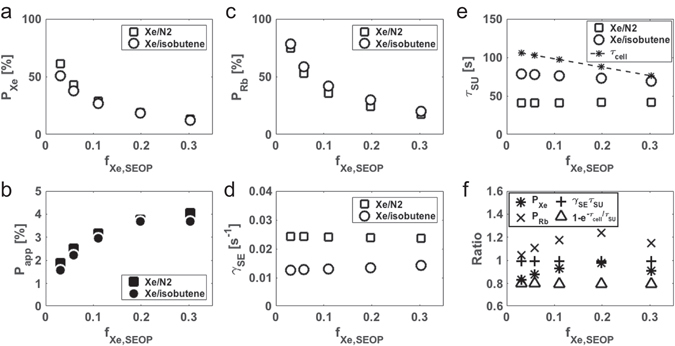
Comparison between isobutene and N_2_ as the quenching gas for Rb-Xe SEOP. Plot of *P*_Xe_ (**a**), *P*_app_ (**b**), *P*_Rb_ (**c**), *γ*_SE_ (**d**), and *τ*_SU_ (**e**) as a function of *f*_Xe,SEOP_ for a Xe/N_2_ mixture (rectangles) and Xe/isobutene mixture (circles) without the separation procedure. The residency time of atoms in SEOP cell, *τ*_cell_, is also shown in (**e**). (**f**) Plot of the ratio of *P*_Xe_, *P*_Rb_, *γ*_SE_ *τ*_SU_, and a factor of $1-{e}^{-{\tau }_{{\rm{cell}}}/{\tau }_{{\rm{SU}}}}$ for isobutene mixture relative to those for N_2_ mixture as a function of *f*_Xe,SEOP_. Gas flow conditions used in these experiments were listed in Table 1.

In order to maximize a benefit from signal enhancement by concentrating HP ^129^Xe, it is important that the use of isobutene did not cause a significant loss of *P*_Xe_ compared with using conventional N_2_. The influence of using isobutene as an alternative quenching gas to conventional N_2_ on *P*_Xe_ was investigated by comparing with that of N_2_. The details of calculations of *P*_Xe_ are given in Methods section. The *P*_Xe_ for each diluted Xe gas mixture (i.e., Xe/isobutene or Xe/N_2_) was measured for the five conditions with the gas mixture. The Xe flow rate (*F*_Xe_) varied from 2.2 to 30 sccm (cubic centimeter per minute at standard temperature and pressure (STP)) whereas that of isobutene (*F*_ib_) or N_2_ $({F}_{{{\rm{N}}}_{2}})$ was kept constant at 69 sccm; therefore, the volume fraction of Xe in the source gas mixture, *f*_Xe,SEOP_, ranged from 0.03 to 0.30 (Table 1). Figure 2a compares *P*_Xe_ between isobutene and N_2_ in the gas mixture without a cold trap. Here, the volume fraction of Xe in the gas mixture at measurement region, *f*_Xe,meas_, was equal to that in the source gas mixture, *f*_Xe,SEOP_. When using the isobutene mixture, *P*_Xe_ was 2–17% lower than that observed with the N_2_ mixture in these experimental conditions. A similar tendency between the two mixtures was observed for the dependence of *P*_Xe_ on *f*_Xe,SEOP_; namely, *P*_Xe_ increased with decreasing *f*_Xe,SEOP_ because the lower Xe number density in SEOP process as is well-known.

**Table 1:** Flow rates and volume fractions of the gas mixture, and partial pressures and number densities of atoms in SEOP cell at a temperature of 383 K and total pressure of 15 kPa.

Flow rate, *F* [sccm]			Fraction, *f*		Partial pressure, *p* [kPa]		Atomic density, *n* [×10^18^ cm^−3^]	
*F*_Xe_	${{\boldsymbol{F}}}_{{{\bf{N}}}_{{\bf{2}}}}$ or *F*_ib_	*F*_tot_	*f*_Xe_	${{\boldsymbol{f}}}_{{{\bf{N}}}_{{\bf{2}}}}$ or *f*_ib_	*p*_Xe_	${{\boldsymbol{p}}}_{{{\bf{N}}}_{{\bf{2}}}}$ or *p*_ib_	*n*_Xe_	${{\boldsymbol{n}}}_{{{\bf{N}}}_{{\bf{2}}}}$ or *n*_ib_
2.2	69.0	71.2	0.03	0.97	0.5	14.5	0.09	2.75
4.3	69.0	73.3	0.06	0.94	0.9	14.1	0.17	2.67
8.5	69.0	77.5	0.11	0.89	1.6	13.4	0.31	2.53
17.0	69.0	86.0	0.20	0.80	3.0	12.0	0.56	2.28
30.0	69.0	99.0	0.30	0.70	4.5	10.5	0.86	1.98

The temperature inside the cell was assumed to be the same as the one of the oven.

In order to compare the ^129^Xe signal intensities in the various experimental conditions, the apparent polarization, *P*_app_, was defined as refs 19 and 20:

1 $${P}_{{\rm{app}}}={P}_{{\rm{Xe}}}\cdot {f}_{{\rm{Xe}},{\rm{meas}}}$$

Details of *P*_app_ are given in Methods section. The *f*_Xe,SEOP_ dependence of *P*_app_ was also observed to have a similar tendency between the two mixtures (Fig. 2b). Owing to a dilution effect, *P*_app_ decreased with decreasing *f*_Xe,SEOP_ even though *P*_Xe_ increased.

The use of isobutene resulted in a slight loss of *P*_Xe_ compared with N_2_. In order to understand how isobutene affects the Rb-Xe SEOP process and clarify the rationale of the polarization loss, we further analyzed the experimental data using a standard model of SEOP and compared several factors affecting *P*_Xe_ between isobutene and N_2_ mixtures. The nuclear spin polarization of ^129^Xe at the output of the SEOP cell in the flow-though polarizer is given by ref. 27

2 $${P}_{{\rm{Xe}}}({\tau }_{{\rm{cell}}})={\gamma }_{{\rm{SE}}}{\tau }_{{\rm{SU}}}\langle {P}_{{\rm{Rb}}}\rangle \,(1-{e}^{-{\tau }_{{\rm{cell}}}/{\tau }_{{\rm{SU}}}})$$

where *γ*_SE_ is the Rb-^129^Xe spin-exchange rate, which includes the contributions of spin exchange induced by Rb-Xe van der Waals molecules, ${\gamma }_{{\rm{SE}}}^{{\rm{vdW}}}$, and spin exchange caused by Rb-Xe binary collisions, ${\gamma }_{{\rm{SE}}}^{{\rm{BC}}}$, according to refs 10 and 27

3 $${\gamma }_{{\rm{SE}}}={\gamma }_{{\rm{SE}}}^{{\rm{vdW}}}+{\gamma }_{{\rm{SE}}}^{BC}=(\frac{1}{{\sum }_{j}({n}_{j}/{\xi }_{j})}+\langle \sigma v\rangle )\,{n}_{{\rm{Rb}}}$$

where *ξ*_*j*_ denotes the van der Waals-specific rate for each gas atom with density *n*_*j*_, and 〈*σv*〉 is the velocity averaged binary spin-exchange cross section. *τ*_SU_ is the spin-up time constant defined as ref. 27

4 $${\tau }_{{\rm{SU}}}^{-1}={\gamma }_{{\rm{SE}}}+{{\rm{\Gamma }}}_{{\rm{Xe}}}$$

where Γ_Xe_ is the ^129^Xe spin relaxation rate, which is though to be dominated by wall relaxation^8, 10^. The wall relaxation rate is pressure independent and depends on the cell geometry (surface/volume) and its surface properties (paramagnetic impurities). 〈*P*_Rb_〉 is the volume-averaged Rb polarization in the cell. *τ*_cell_ is the mean ^129^Xe residence time in the cell, which is related to the total flow rate, *F*_tot_, according to

5 $${\tau }_{{\rm{cell}}}=\frac{{V}_{{\rm{cell}}}}{{F}_{{\rm{tot}}}}\cdot \frac{{p}_{{\rm{cell}}}}{{p}_{{\rm{s}}}}$$

where *V*_cell_ is the cell volume and *p*_cell_ is the total pressure of the gas in the cell. Among the two contributions to the Rb-Xe spin exchange, the formation and break up of Rb/Xe van der Waals molecular complex is affected by the difference in gas atoms or molecules, which is characterized by van der Waals specific rate, *ξ*_*j*_ according to Eq. 3. We could estimate the *ξ* for isobutene as *ξ*_ib_ = 2.61 × 10^3^ s^−1^ (see Methods section) and found that the *ξ*_ib_ is less than half of ${\xi }_{{{\rm{N}}}_{2}}$ (=5.70 × 10^3^ s^−1^ ^28, 29^). The low *ξ* results in low spin exchange rate, *γ*_SE_, which was 40–48% lower for isobutene mixture than for N_2_ mixture under our experimental conditions summarized in Table 1 (Fig. 2d). According to Eq. 2, *P*_Xe_ reaches *γ*_SE_ *τ*_SU_〈Rb〉 after a sufficiently long SEOP time compared to the spin up time, *τ*_SU_. Therefore, if Γ_Xe_ is sufficiently small compared to *γ*_SE_, the difference in *γ*_SE_ has a small influence on *P*_Xe_. In our system, sufficiently small Γ_Xe_ was measured as 1.84 × 10^−4^ s^−1^ (see Methods section). Indeed, the *γ*_SE_ *τ*_SU_ was calculated as 0.986–0.987 for isobutene mixture and 0.992–0.993 for N_2_ mixture; therefore, the effect of the difference between two mixtures is less than 1% (Fig. 2f). The lower *γ*_SE_ prolong the spin up time, *τ*_SU_, and requires longer residency time for *P*_Xe_ to reach sufficiently high value. Since the nuclear spin polarization of ^129^Xe builds up with a time constant of *τ*_SU_ according to Eq. 2, especially for the continuous flow system at a low cell pressure, we need to take into account that the residency time becomes short according to Eq. 5. The spin up time, *τ*_SU_, for isobutene was calculated as raged from 69.4 to 78.4 s and from 40.9 to 41.8 s for N_2_. Although these spin up times for both gas mixtures were shorter than *τ*_cell_ (Fig. 2e), which was ranged from 76.1 to 105.8 s for our experimental condition (*V*_cell_ = 848 cc, *p*_cell_ = 15 kPa, and *F*_tot_ ranging from 71.2 to 99.0 sccm), the SEOP time was insufficient for isobutene compared to N_2_ as a factor of $1-{e}^{-{\tau }_{{\rm{cell}}}/{\tau }_{{\rm{SU}}}}$ for isobutene mixture (0.666–0.741) was ~20% lower than that for N_2_ mixture (0.838–0.925) (Fig. 2f).

Interestingly, in all experimental conditions, 〈*P*_Rb_〉 for isobutene mixture was 4–24% higher than that for N_2_ mixture (Fig. 2c,f). The Rb electron spin polarization at a position *z* in the SEOP cell along the laser propagation direction is given by refs 8, 10 and 27

6 $${P}_{{\rm{Rb}}}\,(z)=\frac{{\gamma }_{{\rm{OP}}}\,(z)}{{\gamma }_{{\rm{OP}}}\,(z)+{{\rm{\Gamma }}}_{{\rm{SD}}}}$$

where *γ*_OP_ is the optical pumping rate, which is determined by the overlap of the frequency- and position-dependent laser intensity profile Φ(*ν*,*z*) and the alkali-metal *D*_1_ absorption cross section *σ*_s_(*ν*) according to

7 $${\gamma }_{{\rm{OP}}}\,(z)=\int \,{\rm{\Phi }}\,(\nu ,z)\,{\sigma }_{{\rm{s}}}\,(\nu )\,d\nu $$

Γ_SD_ is the Rb spin polarization destruction rate, which can be attributed to spin depolarizing binary collisions with atoms in the gas mixture, the formation and breakup of Rb-Xe van der Waals molecules and radiation trapping^19, 30^:

8 $${{\rm{\Gamma }}}_{{\rm{SD}}}=\sum _{j}\,{n}_{j}{k}_{{\rm{SD}}}^{{\rm{Rb}}-j}+{{\rm{\Gamma }}}_{{\rm{SD}}}^{vdW}+{{\rm{\Gamma }}}_{{\rm{SD}}}^{{\rm{trap}}}$$

where ${k}_{{\rm{S}}{\rm{D}}}^{{\rm{R}}{\rm{b}}-j}$ is the Rb spin-destruction cross section for Rb binary collisions with each gas atom with an atomic density *n*_*j*_. Therefore, the Rb polarization is limited by the Rb spin destruction processes characterized by a rate constant of Γ_SD_ (Eq. 6). Since the difference in gases alters all the rate constants involved in these processes, the higher value of 〈*P*_Rb_〉 for isobutene mixture indicates that the isobutene has a valuable property of sufficiently small contribution to the spin destruction of Rb compared to N_2_. The optical pumping rate, *γ*_OP_ includes the effects of pressure broadening of the Rb absorption line and shift of its central line by buffer gases. The pressure-broadening and shift coefficients for Rb have been measured for He, Xe and N_2_ and they are slightly different ranging from 18.0 to 18.9 GHz/amg for broadening and from −8.3 to 5.6 GHz/amg for shift^31^. Although the difference in gases may be less effective for the behavior of broadening and shift as reported for these three gases, the use of isobutene has a possibility to change the *γ*_OP_ compared to the case of N_2_. In order to understand how isobutene affects spin destruction of Rb and optical pumping rate, further study is required, in which important physical quantities such as the absorption line broadening factor and each rate constant related to spin depolarizing binary collisions, van der Waals molecules and radiation trapping should be quantified. At this stage, our results suggested that isobutene seems to act as a quenching gas effectively because observed Rb polarization was higher than that for N_2_ mixture.

In terms of the reactivity of hydrocarbon gases coexisting with highly reactive Rb in the SEOP cell, Rb does not seem to readily react with saturated hydrocarbon gases such as methane^21, 32^, ethane^32^ and butane^33^. Any reaction also seems to be very slow for the unsaturated hydrocarbon isobutene used here. In fact, degradation of the SEOP cell using the isobutene mixture was adequately slow and at an acceptable level to complete *in vivo* experiments with a sufficient number of mice, e.g., 50 mice^25^.

### Dependence of ^129^Xe signal enhancement on the volume fraction of Xe in the diluted gas mixture

**Figure 3:**
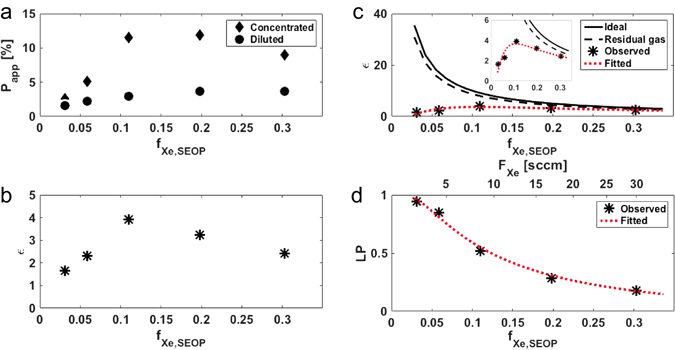
Dependence of ^129^Xe signal enhancement on the Xe fraction in a dilute gas mixture. (**a**) Plot of *P*_app_ as a function of *f*_Xe,SEOP_ for a Xe/isobutene mixture. Data were obtained for a dilute Xe mixture without the separation (circles, the same data as shown in Fig. 2b) and for concentrated Xe after separation from isobutene at 196 K (diamonds). (**b**) Plot of enhancement factor *ε* as a function of *f*_Xe,SEOP_. Gas flow conditions used in these experiments were listed in Table 1. (**c**) Comparison of ^129^Xe signal enhancement between experimental results and ideal conditions. Asterisks: observed enhancement factor, which are the same data as shown in (**b**) solid line: ideal enhancement factor; dashed line: ideal enhancement factor including residual isobutene at 196 K; dotted line: fitted curve using Eq. 11 with *f*_ib,res_ = 0.13. Inset shows the expanded plot of the main figure for clarify. (**d**) Plot of ^129^Xe polarization loss factor, *LP*, as a function of *f*_Xe,SEOP_. The flow rate of Xe is indicated above the plot. The plotted values were calculated using a relation of Eq. 10 with *f*_ib,res_ = 0.13. The dotted line was drawn based on Eq. 13 using the values of *α* estimated from the fit as shown in **c** and *f*_ib,res_ = 0.13.

In the same gas flow conditions as described in the previous section, the enhancement of ^129^Xe signal by removing isobutene was investigated. Figure 3a shows dependence of *P*_app_ on *f*_Xe,SEOP_ measured from concentrated Xe and that from diluted Xe using Xe/isobutene mixtures. Here *f*_Xe,meas_ was different from *f*_Xe,SEOP_ for the concentrated Xe because isobutene was removed from the source gas mixture. For all conditions, the removal of isobutene from Xe gas enabled *P*_app_ to increase (i.e., the enhancement was due to the increase in *f*_Xe,meas_ in Eq. 1). The highest value of *P*_app_ = 11.9% was obtained at *f*_Xe,SEOP_ = 0.20, whereas *P*_app_ = 3.7% was observed for diluted Xe at *f*_Xe,SEOP_ = 0.30.

In order to assess the effectiveness of concentrating Xe from the dilute Xe gas mixture, an enhancement factor, *ε*, was defined as the ratio of the amplitude of ^129^Xe signal obtained from concentrated Xe relative to that from diluted Xe with the same gas mixture used. Details about *ε* are given in the Methods section. The enhancement factor can be expressed by using the index *P*_app_ as:

9 $$\varepsilon =\frac{{P}_{{\rm{app}},{\rm{conc}}}}{{P}_{{\rm{app}},{\rm{dil}}}}$$

where *P*_app,conc_ and *P*_app,dil_ are the apparent polarization observed from concentrated Xe and diluted Xe, respectively. Figure 3b shows dependence of *ε* on *f*_Xe,SEOP_. The maximum enhancement factor of *ε* = 3.9 was observed at *f*_Xe,SEOP_ = 0.11.

Although the ^129^Xe NMR signal was enhanced by the proposed method, the observed enhancement did not reach the value expected. From Eqs 1 and 9, the enhancement factor can be further rewritten as:

10 $$\varepsilon =\frac{{P}_{{\rm{Xe}},{\rm{conc}}}}{{P}_{{\rm{Xe}},{\rm{dil}}}}\cdot \frac{1-{f}_{{\rm{ib}},{\rm{res}}}}{{f}_{{\rm{Xe}},{\rm{SEOP}}}}$$

where *P*_Xe,conc_ and *P*_Xe,dil_ are the polarization for concentrated Xe and diluted Xe, respectively, and *f*_ib,res_ is the fraction of residual isobutene in the gas mixture after the removal process. Note that *P*_Xe,conc_ and *P*_Xe,dil_ are the polarization observed in the measured region (i.e., in the NMR magnet) and, therefore, there is a possibility of a difference between them, although the polarization at the exit of the SEOP cell was the same for both concentrated and diluted conditions. According to Eq. 10, the specific relation *ε* = 1/*f*_Xe,SEOP_ holds true in conditions that satisfy the following two assumptions: (*I*) isobutene is completely removed from the gas mixture by the cold trap (i.e., *f*_ib,res_ = 0), and (*II*) the separation process has no influence on *P*_Xe_ (i.e., *P*_Xe,conc_ = *P*_Xe,dil_). In this ideal condition, it was expected that extremely high *ε* can be obtained at lower *f*_Xe,SEOP_. Figure 3c compares *ε* between the ideal (solid line) and the observed values (asterisks). At the lower *f*_Xe,SEOP_, the observed *ε* was far lower than the ideal one, whereas it was closer to the ideal one with increasing *f*_Xe,SEOP_ (e.g., *ε*~33 and *ε*~1.7 at *f*_Xe,SEOP_ = 0.03 for ideal case and measured value, respectively, whereas *ε*~3.3 and *ε*~2.4 at *f*_Xe,SEOP_ = 0.30 for ideal case and measured value, respectively). The first assumption described above (*I*) was not established in our experiments. *f*_ib,res_ can be estimated from the vapor pressure of isobutene, which is related to the cold trap temperature, and the total pressure at the separation region. The vapor pressure of isobutene at 196 K is ~2.0 kPa^34^, leaving 13% of isobutene in the gas mixture at 15 kPa total pressure, which was our experimental condition. This caused an increase in *f*_ib,res_ from the ideal value of 0 to 0.13, and thus *ε* decreased 13% from the ideal one (Fig. 3c, dashed line). With regard to the second assumption above (*II*), there were three possible causes to induce a difference in *P*_Xe_ between the concentrated and diluted conditions: (*i*) a reduction in ^129^Xe magnetization at the gas–liquid surface in the concentrated condition. The depolarization was expected when gaseous Xe atoms contact with a liquid surface, where an interaction occurs between the ^129^Xe atoms and ^1^H of the dense liquid isobutene molecules. In addition, an exchange phenomenon of Xe atoms between gaseous Xe and depolarized dissolved Xe caused a loss of gaseous Xe magnetization. (*ii*) A difference in relaxation time between concentrated and diluted conditions downstream of the separation region. Because Xe interacts with Xe in concentrated conditions whereas Xe interacts with both Xe and isobutene in diluted condition, the difference in gas composition could cause a difference in relaxation time. (*iii*) A difference in time needed for gas transport from the separation region to the measurement region. The reduced total flow rate due to the removal of isobutene could cause extra depolarization before the measurements. In order to analyze our experimental data in relation to these possibilities, *ε* was rewritten to include relaxation and flow rate:

11 $$\varepsilon =\frac{\exp \,(-{\alpha }_{{\rm{conc}}}/{F}_{{\rm{tot}},{\rm{conc}}})}{\exp \,(-{\alpha }_{{\rm{dil}}}/{F}_{{\rm{tot}},{\rm{dil}}})}\cdot \frac{{F}_{{\rm{t}}ot,dil}}{{F}_{{\rm{t}}ot,conc}}$$

with

$${F}_{{\rm{tot}},{\rm{conc}}}={F}_{{\rm{Xe}}}+{F}_{{\rm{ib}},{\rm{res}}},\,{F}_{{\rm{tot}},{\rm{dil}}}={F}_{{\rm{Xe}}}+{F}_{{\rm{ib}}},\,{\alpha }_{{\rm{conc}}}=\sum _{i}\,\frac{{V}_{i}}{{T}_{1,i,{\rm{conc}}}}\cdot \frac{{p}_{i}}{{p}_{{\rm{s}}}},\,{\alpha }_{{\rm{dil}}}=\sum _{i}\,\frac{{V}_{i}}{{T}_{\mathrm{1,}i,{\rm{dil}}}}\cdot \frac{{p}_{i}}{{p}_{{\rm{s}}}}$$

where the suffixes “dil” and “conc” denote the parameters related to the diluted Xe gas mixture and concentrated Xe gas, respectively, *F*_tot_ is the total flow rate downstream from the separation region, *F*_ib,res_ is the flow rate of residual isobutene after the removal process, *α* is a constant involved in ^129^Xe relaxation, *i* denotes a site in which the relaxation occurs, *T*_1,*i*_ is the ^129^Xe *T*_1_ at site *i*, *V*_*i*_ is the volume at site *i*, and *p*_*i*_ and *p*_s_ are the total pressure of the gas at site *i* and standard-state pressure, respectively. The total flow rate was measured at ambient pressure. The depolarization effects described for (*i*) and (*ii*) were included in *α*_conc_ and *α*_dil_ in Eq. 11 as a difference in the number of sites, where the relaxation is occurred, and difference in the relaxation time. Details about the relationship between Eqs 10 and 11 are given in the Methods section. By fitting the observed *ε* to Eq. 11, *α*_conc_ and *α*_dil_ were estimated as 8.6 and 8.4 sccm, respectively, with a coefficient of determination of *R* ^2^ = 0.733 (Fig. 3c, dotted line). From these values, the degree of relaxation in the concentrated condition could be similar to that in diluted conditions. Here, we introduce a polarization loss factor (*LP*) defined as:

12 $$LP=1-RP$$

where a retained polarization fraction (*RP*) is a factor that describes the fraction of polarization retained when the gas passes through the buffer gas separation procedure and expressed using Eqs 10 and 11 (see also the Methods section for *RP*):

13 $$RP=\frac{{P}_{{\rm{Xe}},{\rm{conc}}}}{{P}_{{\rm{Xe}},{\rm{dil}}}}=\frac{\exp \,(-{\alpha }_{{\rm{conc}}}/{F}_{{\rm{tot}},{\rm{conc}}})}{\exp \,(-{\alpha }_{{\rm{dil}}}/{F}_{{\rm{tot}},{\rm{dil}}})}$$

Figure 3d shows a plot of the *LP* as a function of *f*_Xe,SEOP_ as well as *F*_Xe_. *LP* increased with decreasing *f*_Xe,SEOP_ because of the reduced flow rate for the concentrated condition. Therefore, the discrepancy of *ε* between our experimental results and the ideal case could be mainly attributed to the reduced total flow rate because of the removal of isobutene described in (*iii*).

### Requirements for improving the system

In order to improve the system and produce HP ^129^Xe gas with higher *P*_app_, *LP* needs to be minimized. Based on our results, higher *F*_Xe_ was required to suppress the reduction in *P*_Xe,conc_ (i.e. the increase in *LP*) and attain higher signal enhancement. In fact, it was confirmed experimentally that *ε* was improved by increasing *F*_Xe_ because of the partial suppression of relaxation during the concentrated Xe gas transfer, as shown in Table 2, where the results using a 3% Xe/97% isobutene mixture with different total flow rates of *F*_tot_ = 71.2 and *F*_tot_ = 142.3 sccm (i.e. *F*_Xe_ = 2.2 and *F*_Xe_ = 4.3 sccm, respectively) were compared. In this case, *LP* was improved from 0.94 to 0.88. However the improved *ε*~3.4 was still far lower than that for the ideal value of ~33 because *LP* remained high value. Additionally, the achievable *P*_Xe,dil_ was low for the higher flow rate because of the short residency time of Xe atoms in SEOP cell (*τ*_cell_ = 53 s) compared to the spin up time of this mixture (*τ*_SU_ = 78 s), resulting in low *P*_app_.

**Table 2:** Effect of the total gas flow rate on *P*_Xe_, *P*_app_, and *ε* using 3% Xe/97% isobutene mixture.

Flow rate, *F* [sccm]			Fraction, *f*		*P*_Xe,dil_ [%]	*P*_app_ [%]		*ε*
*F*_Xe_	*F*_ib_	*F*_tot_	*f*_Xe_	*f*_ib_		dil	conc	
2.2	69.0	71.2	0.03	0.97	50.7	1.57	2.61	1.66
4.3	138.0	142.3	0.03	0.97	26.7	0.81	2.72	3.37

In order to further suppress the relaxation effect and obtain higher *P*_app_, it will be necessary to adopt much higher *F*_Xe_ while keeping *f*_Xe,SEOP_ low. By improving SEOP efficiency, the reduction in *P*_Xe_ at the outlet of the SEOP cell caused by the increasing total flow rate may be suppressed. A simple solution could be to introduce spectrally line-narrowed laser diode array (LDA) to improve the absorption efficiency of Rb. The effectiveness of using the line-narrowed LDA to improve *P*_Xe_ has been shown by other group^19^ and by our group as well for our own SEOP system^35^. Another approach to prevent *P*_Xe,conc_ from reduction was to suppress depolarization during HP ^129^Xe gas transfer from the SEOP cell to the measurement region. For this purpose, the material or inner surface coating as well as the shape of all regions from the outlet of the SEOP cell to the measurement region and any effect of the magnetic field that the HP gas is passing through should be taken into account to prolong the relaxation time of ^129^Xe^36–38^. An additional requirement for improving this system is to optimize the temperature of the cold trap. In this study, isobutene was condensed and removed from Xe gas at only a single temperature around 195 K. At this temperature, ~13% of isobutene remained in the gas mixture, resulting in a reduction in the achievable Xe concentration. Lowering the cold trap temperature can reduce the vapor pressure of isobutene and could improve the separation efficiency.

## Conclusion

We have demonstrated the continuous flow production of nearly pure HP ^129^Xe gas concentrated from dilute HP ^129^Xe gas mixture with higher *P*_Xe_, which could be realized using isobutene as a quenching gas in Rb-Xe SEOP. Useful properties of isobutene as a quenching gas for generating HP ^129^Xe was revealed by the comparison of several parameters related to SEOP with conventional N_2_. Utilizing the large difference in boiling point between isobutene and Xe, isobutene could be condensed and removed from HP ^129^Xe/isobutene gas mixture, resulting in enhancement of the ^129^Xe NMR signal owing to the increased concentration of Xe. By optimizing further the system for better performance, this approach could provide a simple means for performing NMR or MRI measurements continuously using HP ^129^Xe with extremely improved sensitivity in a wide range of research fields.

## Methods

### Polarizer

**Figure 4:**
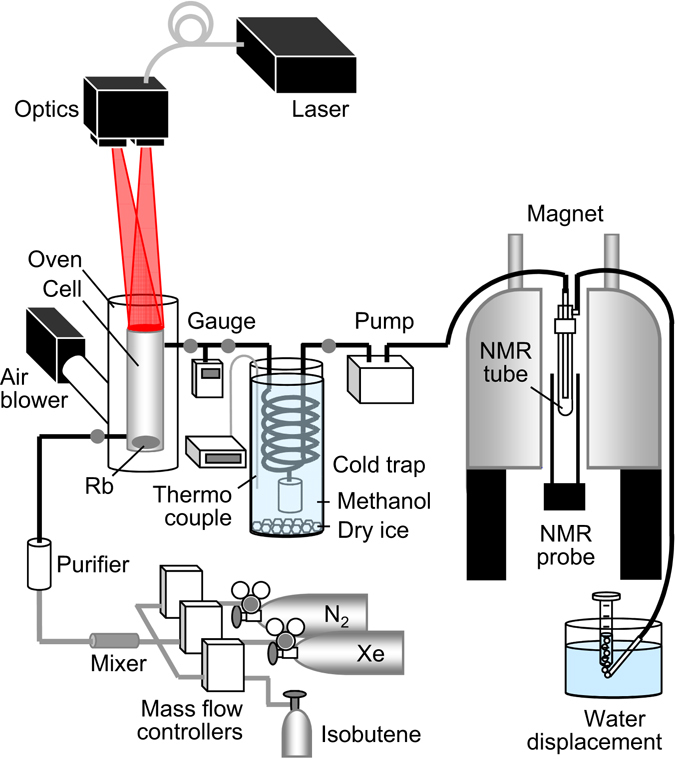
Schematic diagram of the experimental setup. Details are given in the text.

A schematic overview of the experimental setup is shown in Fig. 4, which is a modified system of our low-pressure and flow-through polarizer^26^. Approximately 0.5 g of Rb (EP grade, over 99.5% purity; Nacalai Tesque Inc., Kyoto, Japan) was deposited into a cylindrical Pyrex glass cell (6 cm diameter, 30 cm length). The SEOP cell was placed vertically in a fringe field of 12 mT near a vertical 9.4-T superconducting magnet and housed in an oven. The temperature of the oven was maintained at 383 K using a hot-air blower (CH-6056 HOTWIND S; Leister Technologies AG, Kaegiswil, Switzerland) to increase the Rb vapor pressure. Laser light from two fiber-coupled broadband LDA systems (FAP system, output power ~30 W, linewidth ~2 nm full width at half maximum (FWHM); DUO-FAP system, ~60 W and <6 nm FWHM; COHERENT Inc., CA, USA) were coupled via a transport glass fiber to the circular polarizing unit (COHERENT Inc.). Two circularly polarized beams output from the unit were emitted into the SEOP cell.

Natural abundance Xe (26.44% ^129^Xe; Air Liquide Japan Ltd., Tokyo, Japan), N_2_ (Air Liquide Japan Ltd.), and isobutene (Tokyo Chemical Industry Co., Ltd., Tokyo, Japan) were used. The gas flow rates were controlled independently using individual mass flow controllers (Model 3660, Kojima Instruments Inc, Kyoto, Japan) calibrated for each gas. By adjusting the each flow rate, we controlled the volume fraction of each gas in the gas mixture. Different gases from the outlets of the mass flow controllers were mixed, dried, and then passed into the SEOP cell. The SEOP process was conducted at a pressure of 15 kPa, which was monitored using a pressure gauge (Handy Manometer Model PG-100 102RP, Nidec Copal Electronics Corp., Tokyo, Japan). The resulting HP gas was compressed to atmospheric pressure using a diaphragm pump (LABPORT N86KV.18, KNF Neuberger GmbH, Freiburg, Germany) and delivered into a *ϕ* 10-mm NMR tube placed in an NMR probe via a polyethylene tube (1.4 mm inner diameter, 1.9 mm outer diameter; SP102, Natsume Seisakusho Co., Ltd., Tokyo, Japan). After passing through the NMR tube, the gas flow rate was checked by a water displacement method.

### Gas separation

In order to separate isobutene from the Xe gas, a cold trap was introduced within the system, where isobutene gas was condensed, collected, and stored. The HP gas mixture exiting the SEOP cell was transferred into Pyrex tubular spiral glassware (Fig. 1a), where the gas mixture flowed around and down the spiral, deposited liquid isobutene, and exited up through the vertical return tube. The liquid isobutene dropped down through the spiral and entered a storage vessel placed on the lower side of the spiral. The glassware was cooled by immersing it in dry ice/methanol mixture in a Dewar and maintained at a constant temperature of around 195 K while being monitored using a thermocouple. This temperature was well below the boiling point of isobutene and well above that of Xe (the vapor pressures of isobutene and Xe at 195 K are ~2 kPa and ~428 kPa, respectively), allowing Xe gas to pass through.

Because the solubility of Xe and the relaxation rate of ^129^Xe in organic solvent both increase with decreasing temperature^39, 40^, the separation process was conducted at low pressure to suppress volume loss and polarization loss of ^129^Xe. That is, by reducing the partial pressure of Xe gas, the amount of Xe atoms dissolved into liquid isobutene can be reduced. In addition, shorter residence time of Xe gas in this region can suppress the polarization loss of gaseous ^129^Xe by coming in contact with ^1^H of liquid isobutene at the gas–liquid interface as well as by exchange phenomenon at the liquid surface with dissolved Xe, which relaxed by dipole–dipole interaction with ^1^H of liquid isobutene.

### NMR measurement

All NMR measurements were performed in a vertical 9.4-T superconducting magnet (bore width 89 mm; Oxford Instruments plc., Oxford, UK) with a high-resolution NMR spectrometer, Agilent Unity INOVA 400WB with software VNMR 6.1 C installed (Agilent Technologies, Inc., Santa Clara, CA). A *ϕ* 10-mm NMR probe (Agilent Technologies, Inc.) tuned to the resonant frequency of ^129^Xe (110.6 MHz) was used for detection. The NMR signal of HP ^129^Xe was measured at room temperature with a pulse width of 1 *μ*s, flip angle of 8°, spectral width of 49321.8 Hz, and 16384 data points, without accumulating multiple free induction decay (FID) transients. The FID was recorded repeatedly with a repetition interval of 5 s while the HP gas flowed continuously. The acquired FIDs were phase and baseline corrected and an exponential line broadening of 3 Hz was applied, and then, Fourier transformed to obtain NMR spectra. An SNR was calculated from each spectrum, in which a range from 50 ppm to 100 ppm (with respect to the ^129^Xe spectrum at 0 ppm) was set as the noise region. FWHM of the spectrum, *ν*_FWHM_, was also measured. These processing steps were carried out using VNMR 6.1 C software.

### Polarization

To calculate the *P*_Xe_ of HP ^129^Xe, the signal was compared with that measured for a standard sample of Xe gas at thermal equilibrium according to:

14 $${P}_{{\rm{Xe}},{\rm{p}}}=\frac{{{\rm{SNR}}}_{{\rm{p}}}}{{{\rm{SNR}}}_{{\rm{e}}}}\cdot \frac{{\nu }_{{\rm{FWHM}},{\rm{p}}}}{{\nu }_{{\rm{FWHM}},{\rm{e}}}}\cdot \frac{{f}_{{\rm{Xe}},{\rm{e}}}}{{f}_{{\rm{Xe}},{\rm{p}}}}\cdot \frac{\sqrt{{{\rm{NA}}}_{{\rm{e}}}}}{\sqrt{{{\rm{NA}}}_{{\rm{p}}}}}\cdot {P}_{{\rm{Xe}},{\rm{e}}}$$

where suffixes p and e denote the parameters for hyperpolarized and thermal equilibrium state, respectively, and *f*_Xe_ and NA are the fraction of Xe in the gas mixture and the number of FID accumulation, respectively. As the standard sample, a gas mixture of 50% Xe/50% O_2_ was enclosed at atmospheric pressure within a *ϕ* 10-mm NMR tube. O_2_ was included to promote the efficiency of FID accumulation by reducing ^129^Xe *T*_1_^41^. For the thermal equilibrium gas, 10,000 FID transients were accumulated with the same acquisition parameters as HP ^129^Xe except for a repetition interval of 3 s. Post-processing of the FID was performed in the same manner as HP ^129^Xe. ^129^Xe polarization at thermal equilibrium was calculated from Boltzmann’s distribution law for nuclei with spin *I* = 1/2 according to ref. 42:

15 $${P}_{{\rm{Xe}},{\rm{e}}}\approx \frac{\hslash |\gamma |{B}_{0}}{2{k}_{{\rm{B}}}T}$$

where *ħ* = *h*/2*π*, *γ*, *B*_0_, *k*_B_ and *T* are Plank constant, gyromagnetic ratio of ^129^Xe nuclei, static magnetic field strength (*B*_0_ = 9.4 T), Boltzmann constant, and temperature (typically *T* = 292 K), respectively. For HP ^129^Xe, ten *P*_Xe,p_ calculated from repeatedly acquired spectra were averaged to give final values.

### Enhancement factor

In order to assess the effectiveness of concentrating Xe from a dilute Xe gas mixture, an enhancement factor, *ε*, was defined as the ratio of the amplitude of the ^129^Xe signal, *A*, obtained from concentrated Xe relative to that from diluted Xe with the same gas mixture used. *ε* was calculated from SNR and *ν*_FWHM_ of ^129^Xe spectra according to:

16 $$\varepsilon =\frac{{A}_{{\rm{conc}}}}{{A}_{{\rm{dil}}}}=\frac{{{\rm{SNR}}}_{{\rm{conc}}}}{{{\rm{SNR}}}_{{\rm{dil}}}}\cdot \frac{{\nu }_{{\rm{FWHM}},{\rm{conc}}}}{{\nu }_{{\rm{FWHM}},{\rm{dil}}}}$$

where the suffixes “dil” and “conc” denote the parameters related to experiments with diluted Xe gas mixture without a separation procedure and experiments with concentrated Xe gas by removing isobutene using a cold trap, respectively. The amplitude of the ^129^Xe NMR signal obtained from a Xe gas mixture was proportional to the ^129^Xe polarization, *P*_Xe_, and the number density of ^129^Xe in the gas mixture at the measurement region, *n*_Xe,meas_. In the present study, because the signal was measured at constant temperature and atmospheric pressure, *n*_Xe,meas_ was only the function of a fraction of Xe in the gas mixture at the measurement region, *f*_Xe,meas_, and therefore:

17 $$A\propto {P}_{{\rm{Xe}}}\cdot {f}_{{\rm{Xe}},{\rm{meas}}}$$

For the diluted condition, *f*_Xe,meas_ was equal to the fraction of Xe in the gas mixture in the SEOP region, *f*_Xe,SEOP_, whereas for the concentrated condition, *f*_Xe,meas_ becomes 1 − *f*_ib,res_ with *f*_ib,res_ as a fraction of residual isobutene in the gas mixture after the removal process. Therefore, the enhancement factor can be expressed as Eq. 10.

### Apparent polarization

In order to compare the ^129^Xe signal intensities between diluted and concentrated conditions for various gas mixtures, the fraction of Xe in the gas mixture needs to be taken into account together with the polarization of ^129^Xe^17, 19, 20^. Apparent polarization, *P*_app_, has been proposed by Meersmann and coworkers^19, 20^ in this regard as Eq. 1. *P*_app_ is a measure of the polarization taking into account the fraction of Xe in the gas mixture and scaled to the polarization in pure HP ^129^Xe gas, allowing direct comparison of the signal intensity of dilute Xe gas mixture with that of pure Xe gas at polarization *P*_Xe_. By using the *P*_app_, the enhancement factor is also expressed as the ratio of apparent polarization as Eq. 9. The apparent polarization for the diluted condition, *P*_app,dil_, was calculated from a measured *P*_Xe,dil_ and a setup condition of *f*_Xe,SEOP_ as:

18 $${P}_{{\rm{app}},{\rm{dil}}}={P}_{{\rm{Xe}},{\rm{dil}}}\cdot {f}_{{\rm{Xe}},{\rm{SEOP}}}$$

whereas that for the concentrated condition, *P*_app,conc_, was calculated from *P*_app,dil_ and *ε* using relations of Eqs 10 and 18 as:

19 $${P}_{{\rm{app}},{\rm{conc}}}={P}_{{\rm{Xe}},{\rm{conc}}}\cdot \mathrm{(1}-{f}_{{\rm{ib}},{\rm{res}}})={P}_{{\rm{app}},{\rm{dil}}}\cdot \varepsilon $$

because *P*_Xe,conc_ cannot be determined due to uncertainty of *f*_ib,res_.

### Retained polarization fraction

In the experimental setup used in this study, Xe gas mixture polarized at the SEOP cell was transferred to the NMR tube in which the ^129^Xe NMR signal is measured. During this transfer process, the nuclear spin polarization of ^129^Xe was depolarized by various factors. This polarization loss was simply defined here as:

20 $${P}_{{\rm{Xe}},{\rm{meas}}}={P}_{{\rm{Xe}},{\rm{SEOP}}}\cdot \exp \,(-\sum _{i}\,\frac{{\tau }_{i}}{{T}_{\mathrm{1,}i}})$$

where *P*_Xe,meas_ and *P*_Xe,SEOP_ are ^129^Xe polarization at the measurement region and at the exit of the SEOP cell, respectively, *i* denotes a site in which the relaxation occurs, *T*_1,*i*_ and *τ*_*i*_ are the longitudinal relaxation time of ^129^Xe at site *i* and the residence time of the gas mixture at site *i*, respectively. *τ*_*i*_ is related to the total gas flow rate at STP, *F*_tot_, according to:

21 $${\tau }_{i}=\frac{{V}_{i}}{{F}_{{\rm{tot}}}}\cdot \frac{{p}_{i}}{{p}_{{\rm{s}}}}$$

where *V*_*i*_ is the volume at site *i*, and *p*_*i*_ and *p*_s_ are the total pressure of the gas at site *i* and standard-state pressure, respectively. The total gas flow rate is the sum of each flow rate for constituents included in the gas mixture, *F*_*j*_:

22 $${F}_{{\rm{tot}}}=\sum _{j}\,{F}_{j}$$

The fraction of each constituent in the gas mixture was defined as:

23 $${f}_{j}=\frac{{F}_{j}}{{F}_{{\rm{tot}}}}$$

When the polarized gas mixture was transferred to the measurement region without separation procedure, the fraction of Xe in the gas mixture at the measurement region, *f*_Xe,meas_, was equal to that at the SEOP region, *f*_Xe,SEOP_:

24 $${f}_{{\rm{Xe}},{\rm{meas}}}={f}_{{\rm{Xe}},{\rm{SEOP}}}=1-\sum _{k}\,{f}_{k,{\rm{SEOP}}}$$

where *f*_*k*,SEOP_ is the fraction of buffer gases in the SEOP region. If the buffer gases were removed after the SEOP process, *f*_Xe,meas_ would increase with the following relation:

25 $${f}_{{\rm{Xe}},{\rm{meas}}}=1-\sum _{k}\,{f}_{k,\mathrm{res}}$$

where *f*_*k*,res_ is the fraction of residual buffer gases in the mixture after the removal process, and has a value ranging from 0 to *f*_*k*,SEOP_. That is, if the buffer gases are removed completely from Xe gas, *f*_Xe,meas_ becomes unity, whereas if the buffer gases are not removed at all, *f*_Xe,meas_ = *f*_Xe,SEOP_, which is the same as for the case described in Eq. 24. A retained polarization factor (*RP*) was defined as a polarization obtained from concentrated Xe, *P*_Xe,meas,conc_, divided by that from diluted Xe mixture, *P*_Xe,meas,dil_. This index means a factor that describes the fraction of polarization retained when the gas passes through the buffer gas separation procedure, and is expressed by using relations of Eqs 20 and 21 as:

26 $$RP=\frac{{P}_{{\rm{Xe}},{\rm{meas}},{\rm{conc}}}}{{P}_{{\rm{Xe}},{\rm{meas}},{\rm{dil}}}}=\frac{\exp \,(-{\alpha }_{{\rm{conc}}}/{F}_{{\rm{tot}},{\rm{conc}}})}{\exp \,(-{\alpha }_{{\rm{dil}}}/{F}_{{\rm{tot}},{\rm{dil}}})}$$

with:

$${\alpha }_{{\rm{conc}}}=\sum _{i}\,\frac{{V}_{i}}{{T}_{1,i,{\rm{conc}}}}\cdot \frac{{p}_{i}}{{p}_{{\rm{s}}}},\quad {\alpha }_{{\rm{dil}}}=\sum _{i}\,\frac{{V}_{i}}{{T}_{1,i,{\rm{dil}}}}\cdot \frac{{p}_{i}}{{p}_{{\rm{s}}}}$$

Here we assumed that the *P*_Xe,SEOP_ is the same value regardless of the use of the cold trap, because the SEOP process, and hence *P*_Xe,SEOP_, was assumed not to be affected by the separation process in the experiments. Therefore, from Eqs 23 to 26, Eq. 10 can be further rewritten as Eq. 11.

### Estimation of van der Waals-specific rate for isobutene

**Figure 5:**
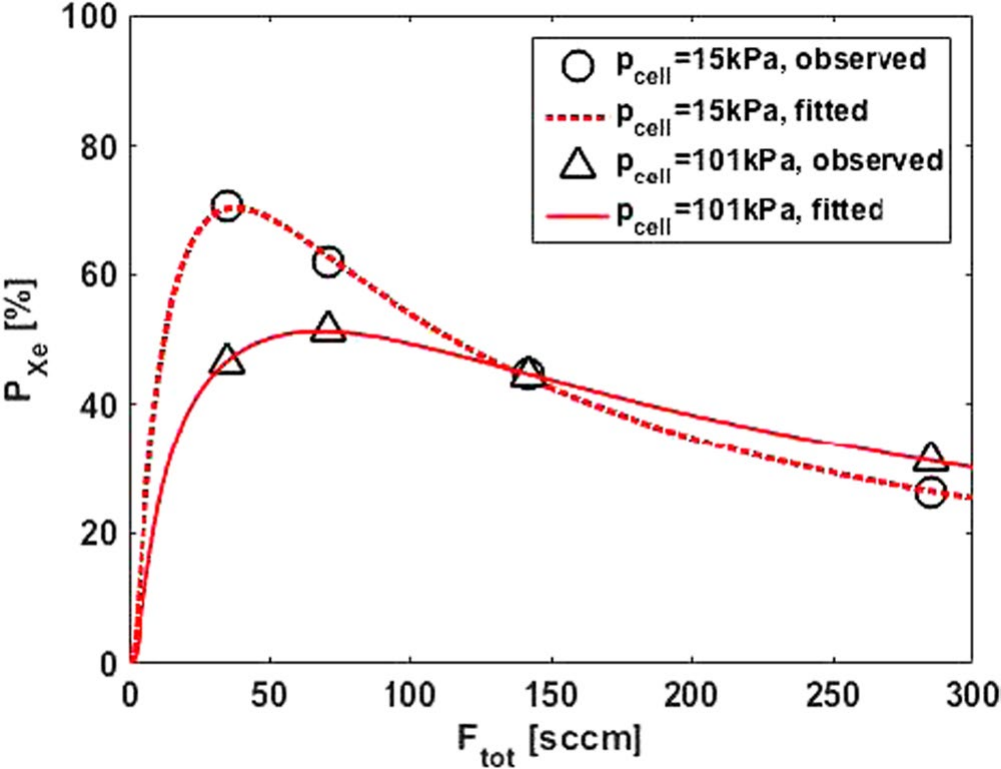
Dependence of *P*_Xe_ on total gas flow rate using 3% Xe/97% isobutene mixture polarized at 15 kPa and 101 kPa. The experiments were performed without a separation procedure. The solid and dashed lines are the result of the fit using Eq. 27. The coefficient of determination of the fit was *R* ^2^ = 0.9991 for 15 kPa and 0.9998 for 101 kPa.

In order to estimate a van der Waals-specific rate for isobutene, *ξ*_ib_, we conducted the following experiments. By using a gas mixture of single composition, 3% Xe/97% isobutene, the total flow rate dependence of *P*_Xe_ were measured for the two pressures in SEOP cell of *p*_cell_ = 15 kPa and *p*_cell_ = 101 kPa. The total flow rate was varied ranging from 35.1 sccm to 285 sccm. The results were analyzed by using following equation. Including a relaxation effect during the transfer process from the output of the SEOP cell to the measurement region into Eq. 2 (see also Retained polarization section), we can express the ^129^Xe polarization at the measurement region as a function of total flow rate as follows:

27 $${P}_{{\rm{Xe}},{\rm{meas}}}\,({F}_{{\rm{tot}}})={P}_{0}\,(1-{e}^{-\beta /{F}_{{\rm{tot}}}})\,{e}^{-\alpha /{F}_{{\rm{tot}}}}$$

with:

$${P}_{0}={\gamma }_{{\rm{SE}}}{\tau }_{{\rm{SU}}}\langle {P}_{{\rm{Rb}}}\rangle ,\quad \beta =\frac{{V}_{{\rm{cell}}}}{{\tau }_{{\rm{SU}}}}\cdot \frac{{p}_{{\rm{cell}}}}{{p}_{{\rm{s}}}},\quad \alpha =\sum _{i}\,\frac{{V}_{i}}{{T}_{\mathrm{1,}i}}\cdot \frac{{p}_{i}}{{p}_{{\rm{s}}}}$$

where *α* is the same definition as the one described in Eq. 26. The experimental data were fitted by using Eq. 27 with *P*_0_ and ${\tau }_{{\rm{SU}}}^{-1}$ as fitting parameters. The *α* was also set as a fitting parameter for the data obtained from the experimental data at *p*_cell_ = 101 kPa whereas *α* = 8.4 sccm was used for that at *p*_cell_ = 15 kPa, which was measured as *α*_dil_ as described in the previous section. The values of *V*_cell_ = 848 cc was used in the analysis. The observed *P*_Xe,meas_ and fitted curves are shown in Fig. 5, and the estimated values from the fit are listed in Table 3 (upper). The van der Waals-specific rate for isobutene and ^129^Xe spin relaxation rate can be calculated from the values of ${\tau }_{{\rm{SU}}}^{-1}$ for two cell pressures using relations of Eqs 3 and 4. In the calculation, literature values of *ξ*_Xe_ and 〈*σv*〉, and the number densities of atoms calculated for our experimental condition listed in Table 3 (lower) were used. The estimated values were *ξ*_ib_ = 2.61 × 10^3^ s^−1^ and Γ_Xe_ = 1.84 × 10^−4^ s^−1^.

**Table 3:** Parameters estimated from the analysis of the total gas flow dependence of *P*_Xe_ using 3% Xe/97% isobutene mixture polarized at 15 kPa and 101 kPa (upper).

Symbol *p*_cell_	*P*_0_ [%]		${{\boldsymbol{\tau }}}_{{\bf{SU}}}^{-{\bf{1}}}$ [s^−1^]		*α* [sccm]	*ξ*_ib_ [s^−1^]	Γ_Xe_ [s^−1^]
	15 kPa	101 kPa	15 kPa	101 kPa	101 kPa		
Value	95.1	62.7	1.28 × 10^−2^	4.06 × 10^−3^	10.4	2.61 × 10^3^	1.84 × 10^−4^
Symbol *p*_cell_	*n*_Xe_ [cm^−3^]		*n*_ib_ [cm^−3^]		*n*_Rb_ [cm^−3^]	*ξ*_Xe_ [s^−1^]	〈*σv*〉 [cm^−3^ s^−1^]
	15 kPa	101 kPa	15 kPa	101 kPa			
Value	8.51 × 10^16^	5.75 × 10^17^	2.75 × 10^18^	1.86 × 10^19^	1.09 × 10^13^ ^43^	5.23 × 10^3^ ^44^	2.17 × 10^−16^ ^45^

Number densities of atoms and spin exchange parameters used in the estimation of *ξ*_ib_ and Γ_Xe_ (lower). The number densities were calculated for 3% Xe/97% isobutene mixture in SEOP cell at a temperature of 383 K and total pressure of 15 kPa and 101 kPa.

### Data Availability

All data generated or analysed during this study are included in this published article.
